# Synthesis, crystal structure, Hirshfeld surface analysis, and energy framework of bis­{3-(4-bromo­phen­yl)-5-[6-(1*H*-pyrazol-1-yl)pyridin-2-yl]-4*H*-1,2,4-triazol-4-ido}nickel(II) methanol disolvate and comparison with its chloro-substituted analogue

**DOI:** 10.1107/S2056989025007467

**Published:** 2025-09-05

**Authors:** Kateryna Znovjyak, Sergiu Shova, Sergiy O. Nikitin, Yurii S. Moroz, Oksana Tananaiko, Sergey O. Malinkin, Maksym Seredyuk

**Affiliations:** a Department of Chemistry, Taras Shevchenko National University of Kyiv, Volodymyrska Street 64, Kyiv, 01601, Ukraine; b Department of Inorganic Polymers "Petru Poni" Institute of Macromolecular Chemistry Romanian Academy of Science Aleea Grigore Ghica Voda 41-A Iasi 700487 Romania; c ChemBioCenter Kyiv National Taras Shevchenko University Kyiv 02094 61 Winston Churchill Street Ukraine; Universidade Federal do ABC, Brazil

**Keywords:** crystal structure, nickel(II) complexes, neutral complexes

## Abstract

The unit cell of the title compound, [Ni(C_16_H_10_BrN_6_)_2_]·2CH_3_OH, contains a neutral complex and two methanol mol­ecules. The Ni^II^ ion adopts a pseudo­octa­hedral geometry, coordinated by two tridentate ligands *via* pyrazole, pyridine, and triazole N atoms. The average Ni—N bond length is 2.097 (4) Å. In the crystal, mol­ecules form supra­molecular chains through weak C–H⋯π inter­actions and further assemble into diperiodic layers *via* C—H⋯N/C inter­actions. Hirshfeld surface analysis shows H⋯H (32.1%), H⋯C/C⋯H (27.3%), H⋯N/N⋯H (14.9%), and H⋯Br/Br⋯H (14.6%) contacts. Inter­action energies were evaluated using HF/3–21 G energy frameworks analysis. Structural parameters were compared to those of the chloro-containing analogue, and the effect of substituent variation was discussed.

## Abstract 2

The title compound, a neutral bis­{3-(4-bromo­phen­yl)-5-[6-(1*H*-pyrazol-1-yl)pyridin-2-yl]-4*H*-1,2,4-triazol-4-ido}nickel(II) methanol disolvate, exhibits a distorted pseudo­octa­hedral coordination environment around the metal ion. Due to the conical geometry and polar characteristics the mol­ecules stack in one-dimensional columns that are connected by weak hydrogen bonds to form layers. These layers are arranged in a three-dimensional lattice without inter­layer inter­actions closer than van der Waals distances.

## 1. Chemical context

A significant category of coordination compounds comprises 3*d*-metal complexes coordinated with tridentate bis­azole­pyridine ligands (Halcrow *et al.*, 2019 ▸; Suryadevara *et al.*, 2022 ▸), which have been employed in diverse applications including catalysis (Xing *et al.*, 2014 ▸; Wei *et al.*, 2015 ▸) and mol­ecular magnetism (Suryadevara *et al.*, 2022 ▸). Recently, we reported an Ni^II^ complex incorporating an asymmetric deprotonated chloro-substituted ligand, 3-(4-chloro­phen­yl)-5-[6-pyrazol­yl(2-pyrid­yl)]-1*H*-1,2,4-triazole (KULRIW; Znovjyak *et al.*, 2024 ▸).
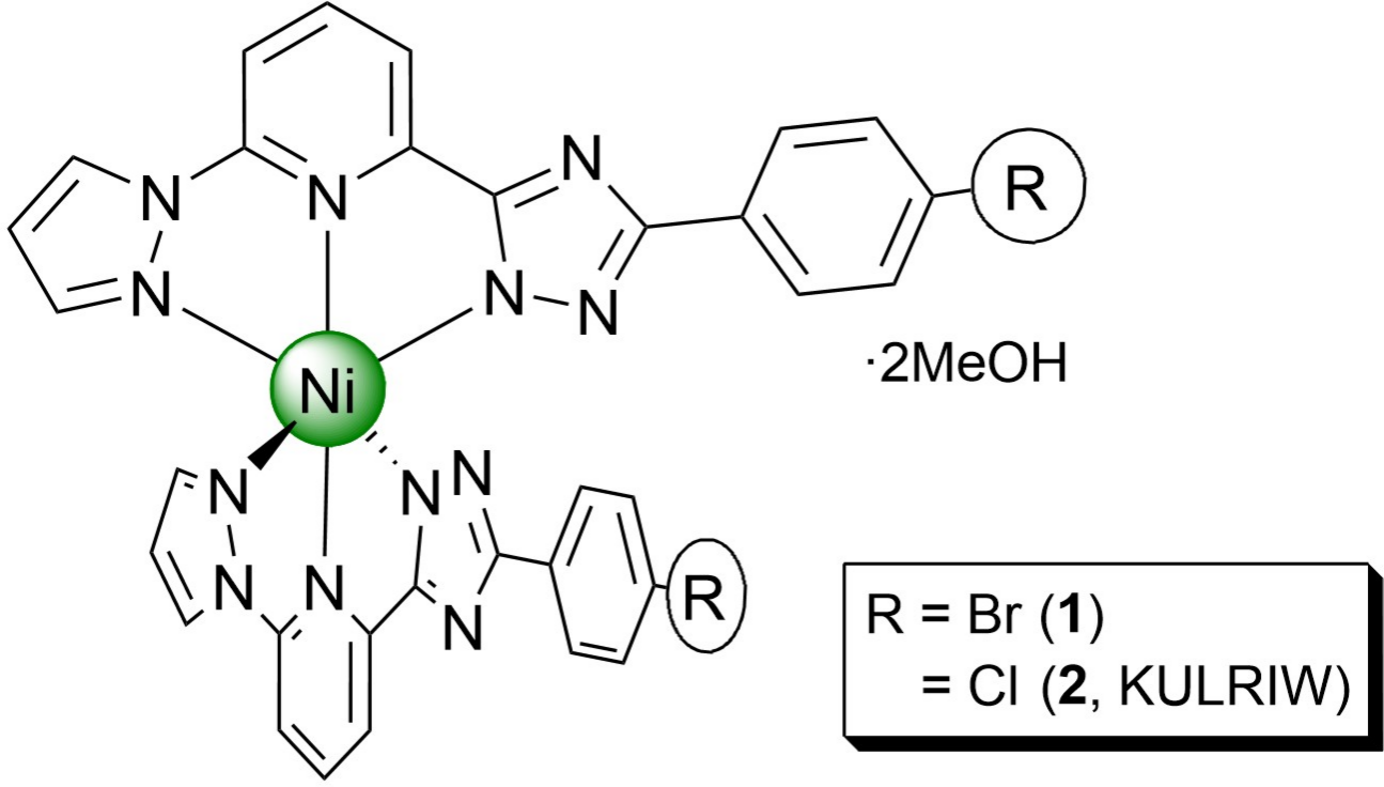


In this study, we describe the synthesis and crystal structure determination of a new complex (**1**) featuring a bromo-substituted ligand, 3-(4-bromo­phen­yl)-5-(6-pyrazol­yl(2-pyrid­yl))-1*H*-1,2,4-triazole. Comprehensive structural analyses were performed and the resulting calculated parameters were compared with those of the chloro-derivative (**2**).

## 2. Structural commentary

**Figure 1:**
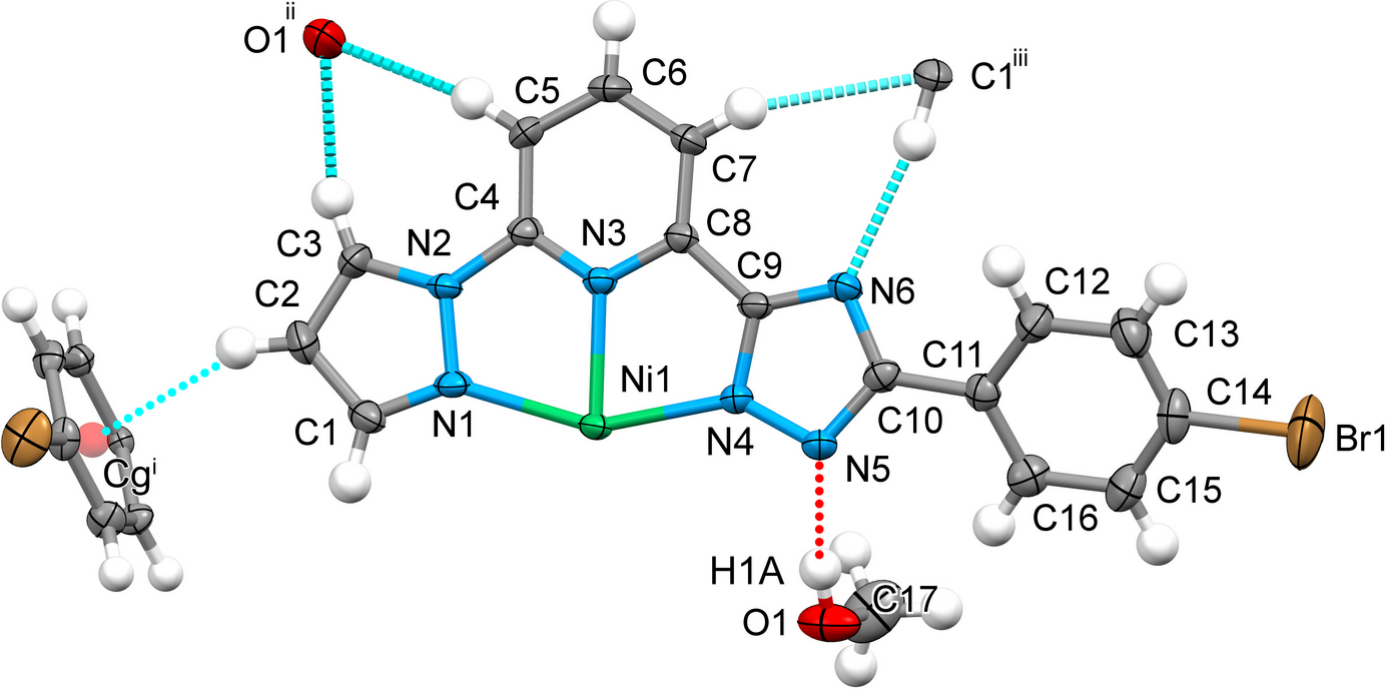
The mol­ecular structure in the asymmetric unit of the title compound and contact atoms with displacement ellipsoids drawn at the 50% probability level. The strong O—H⋯N (red) and weak C—H⋯N/C/O/*C*g (cyan) hydrogen bonds are shown with the nearest neighbors. Symmetry codes: (i) 1 − *x*, 1 + *y*, 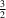 − *z*; (ii) 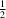 + *x*, 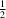 + *y*, 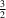 − *z*; (iii) 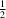 + *x*, −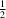 + *y*, 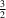 − *z*.

The two tridentate ligands span meridional and perpendicular coordination sites on the octa­hedron, forming a mol­ecule with a compact coordination part and pending diverging 4-bromo­phenyl groups. The pendant group is tilted by 26.6 (2)° relative to the nearly planar pyrazole-pyridine-triazole (pz-py-trz) fragment (r.m.s. deviation = 0.074 Å). A methanol mol­ecule forms an O—H⋯N5 hydrogen bond with the triazole ring of the ligand (Table 1 ▸, Fig. 1 ▸). The central Ni ion adopts a distorted octa­hedral N_6_ coordination sphere, formed by nitro­gen atoms from two tridentate ligands, with an average Ni—N bond length of 2.097 (4) Å. The [NiN_6_] coordination polyhedron has a volume of 11.616 Å^3^. The trigonal distortion parameters are *Σ* = 119.3° (*Σ* = Σ_1_^12^(|90 – *φ*_i_|), where *φ*_i_ is the N—Ni—N′ angle; Drew *et al.*, 1995 ▸) and *Θ* = 386.9° (*Θ* = Σ_1_^24^(|60 – *θ*_i_|), where *θ*_i_ is the angle from superposed opposite octa­hedral faces; Chang *et al.*, 1990 ▸), indicating deviation from ideal octa­hedral geometry (*Σ* = *Θ* = 0). The continuous shape measure [CShM(*O*_h_)] relative to ideal octa­hedral symmetry is 3.702 (Kershaw Cook *et al.*, 2015 ▸). Compared to **2**, compound **1** shows marginally higher distortion indices, reflecting the effect of varying pendant substituents (Table 2 ▸).

## 3. Supra­molecular features

**Figure 2:**
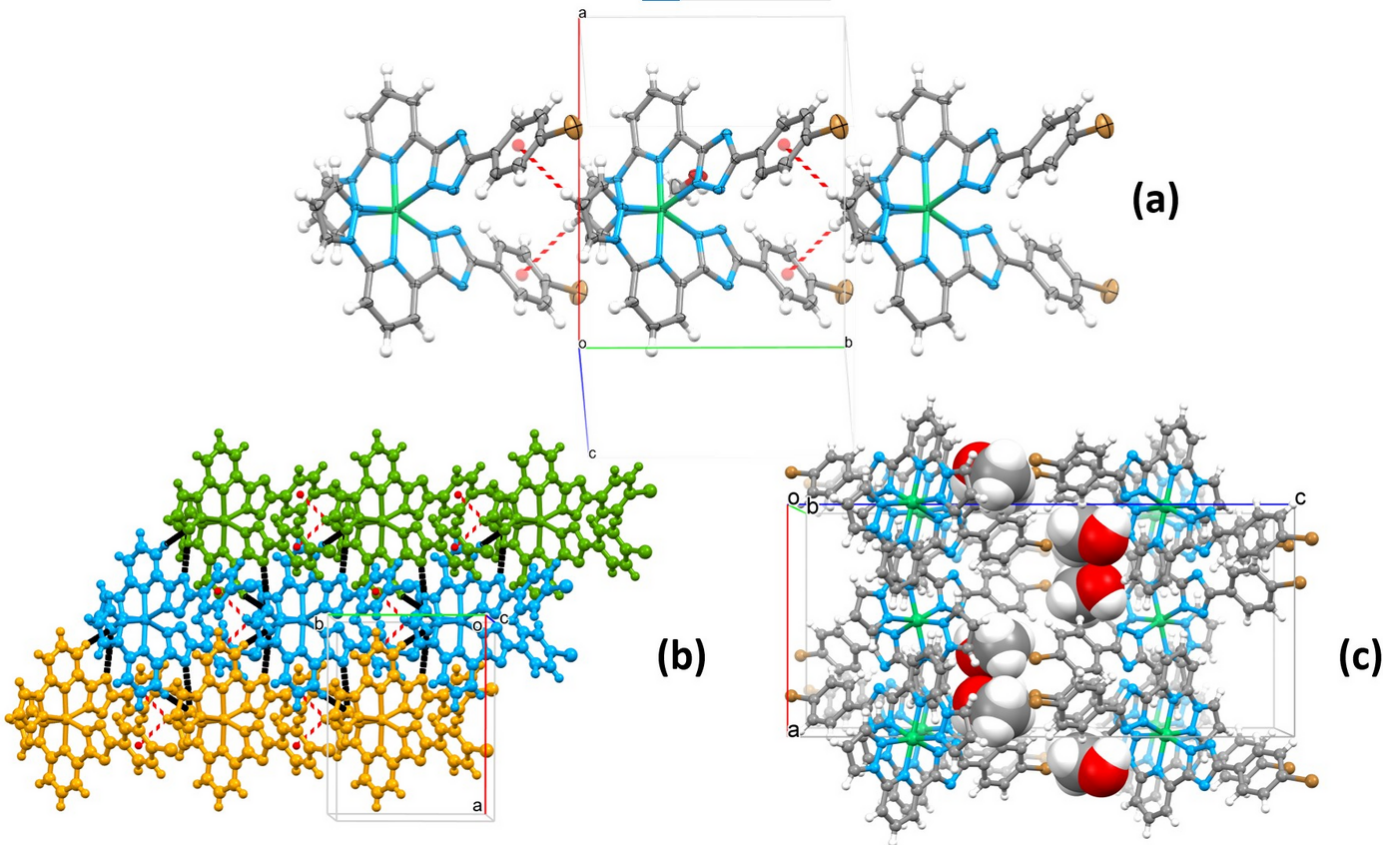
(*a*) A fragment of a monoperiodic supra­molecular column formed by stacking of mol­ecules along the *b* axis, with C—H⋯*Cg* contacts indicated by red dashed lines; (*b*) supra­molecular diperiodic layers formed by stacking supra­molecular columns in the *ab* plane. The C—H⋯N/C contacts between chains are indicated by black dashed cylinders. For a better representation, each column has a different color; (*c*) stacking of the diperiodic layers along the *c* axis with the methanol mol­ecules in the voids.

The title compound exhibits a packing similar to **2**, with adjacent mol­ecules inter­locked and inter­acting *via* weak off-center non-perpendicular (73.0° angle) C—H(pz)⋯π(ph) contact between the pyrazole and phenyl groups [H2/C2⋯*Cg*(ph) = 2.686 (1)/3.542 (6) Å]. The formed one-dimensional chains extend along the *b-*axis direction with a periodicity of 10.1729 (4) Å (Fig. 2 ▸*a*), and are linked into corrugated layers in the *ab* plane by weak C—H(pz, py)⋯N/C(pz, trz) inter­actions [3.238 (6)–3.746 (7) Å; Fig. 2 ▸*b*]. The layers stack without inter­actions below the van der Waals radii, while methanol mol­ecules occupy the inter­layer voids and connect them through weak O⋯H—C(pz,py) inter­actions (Fig. 2 ▸*c*). Table 1 ▸ provides a summary of all inter­molecular inter­actions. Compared to **2**, the overall packing remains similar, with minor differences in the values of inter­molecular contacts, which can be compared using Hirshfeld surface analysis.

## 4. Hirshfeld surface and two-dimensional fingerprint plots

**Figure 3:**
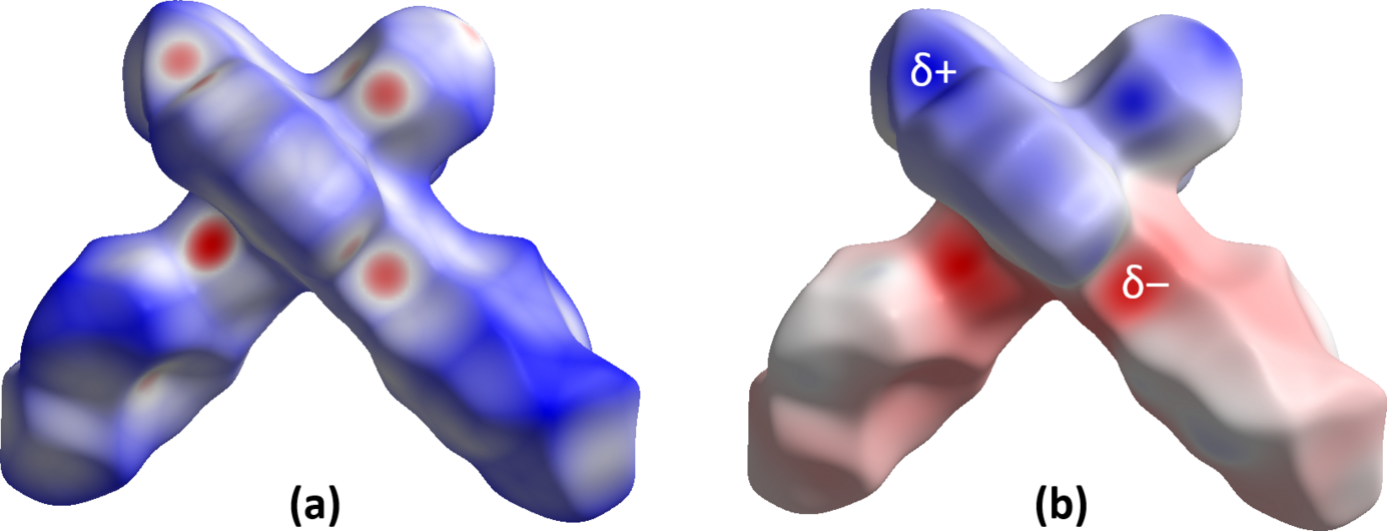
(*a*) A projection of *d*_norm_ mapped on Hirshfeld surfaces, showing the inter­molecular inter­actions within the mol­ecule. Red/blue and white areas represent regions where contacts are shorter/larger than the sum and close to the sum of the van der Waals radii, respectively. (*b*) Electrostatic potential for the title compound mapped on the Hirshfeld surface. Red/blue and white areas represent regions where the charge is negative/positive or close to zero.

**Figure 4:**
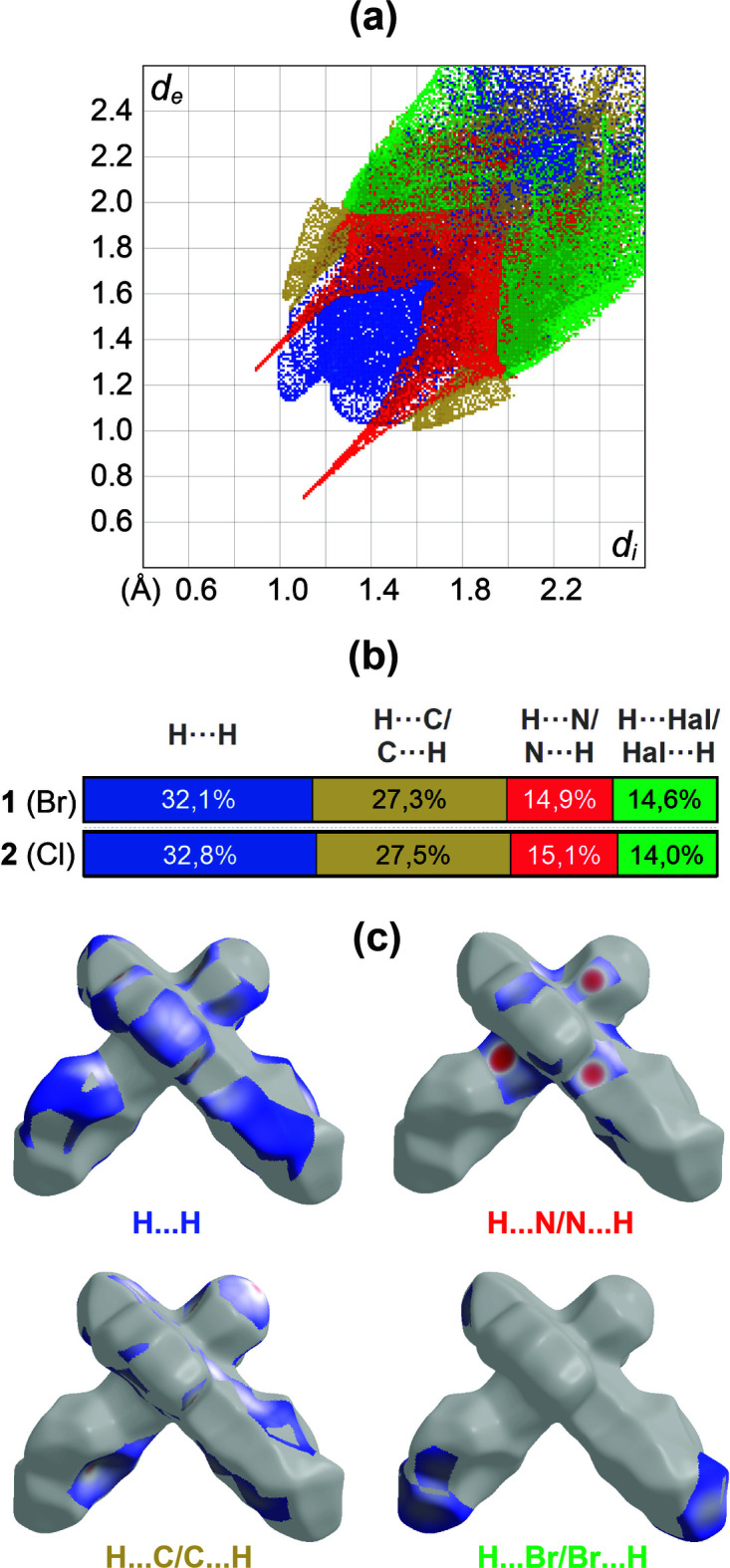
(*a*) Decomposition of the two-dimensional fingerprint plot of **1** into specific inter­actions and (*b*) comparison with those in **2**; (*c*) a projection of *d*_norm_ mapped on the Hirshfeld surfaces, showing the inter­molecular inter­actions within the mol­ecule. Red/blue and white areas represent regions where contacts are shorter/larger than the sum and close to the sum of the van der Waals radii, respectively.

A Hirshfeld surface analysis was conducted and two-dimensional fingerprint plots were generated using *CrystalExplorer 21.5* (Spackman *et al.*, 2021 ▸), with a standard resolution for the three-dimensional *d*_norm_ surfaces plotted over a fixed color scale ranging from −0.6304 (red) to 1.6516 (blue) a.u. Red spots indicate short contacts and negative *d*_norm_ values on the surface, which correspond to the inter­actions described above. A projection of *d*_norm_ mapped over the Hirshfeld surfaces is presented in Fig. 3 ▸*a*. The two-dimensional fingerprint plots, along with their relative contributions to the Hirshfeld surface mapped over *d*_norm_, are shown in Fig. 4 ▸*a*. H⋯H inter­actions account for the largest contribution to the overall crystal packing at 32.1%, and are situated in the middle region of the fingerprint plot. H⋯C/C⋯H contacts contribute 27.3%, while H⋯N/N⋯H contacts, seen as a pair of sharp spikes, represent a 14.9% contribution to the surface. Inter­actions of H⋯Br/Br⋯H make up 14.6%, forming pairs of characteristic wings. This is greater than the H⋯Cl/Cl⋯H inter­action in **2**, while other contributions are smaller due to the larger van der Waals radius of Br compared to Cl (1.85 *vs* 1.75 Å; Bondi, 1964 ▸) and the corresponding relative contribution to the surface area of the mol­ecule. In Fig. 4 ▸*b*, the percentage contribution of contacts to the Hirshfeld surface for the two compounds is compared. In Fig. 4 ▸*c*, the different inter­actions are plotted onto the Hirshfeld surface. The electrostatic potential energy calculated using the HF/3-21G basis is shown in Fig. 3 ▸*b*. The negative charge is localized on the trz-ph moiety and the Br atom of the complex mol­ecule, whereas the pz-py moieties exhibit relatively positive charges, supporting the stacking of mol­ecules into columns and the arrangement of these columns into diperiodic two-dimensional layers.

## 5. Energy framework analysis

**Figure 5:**
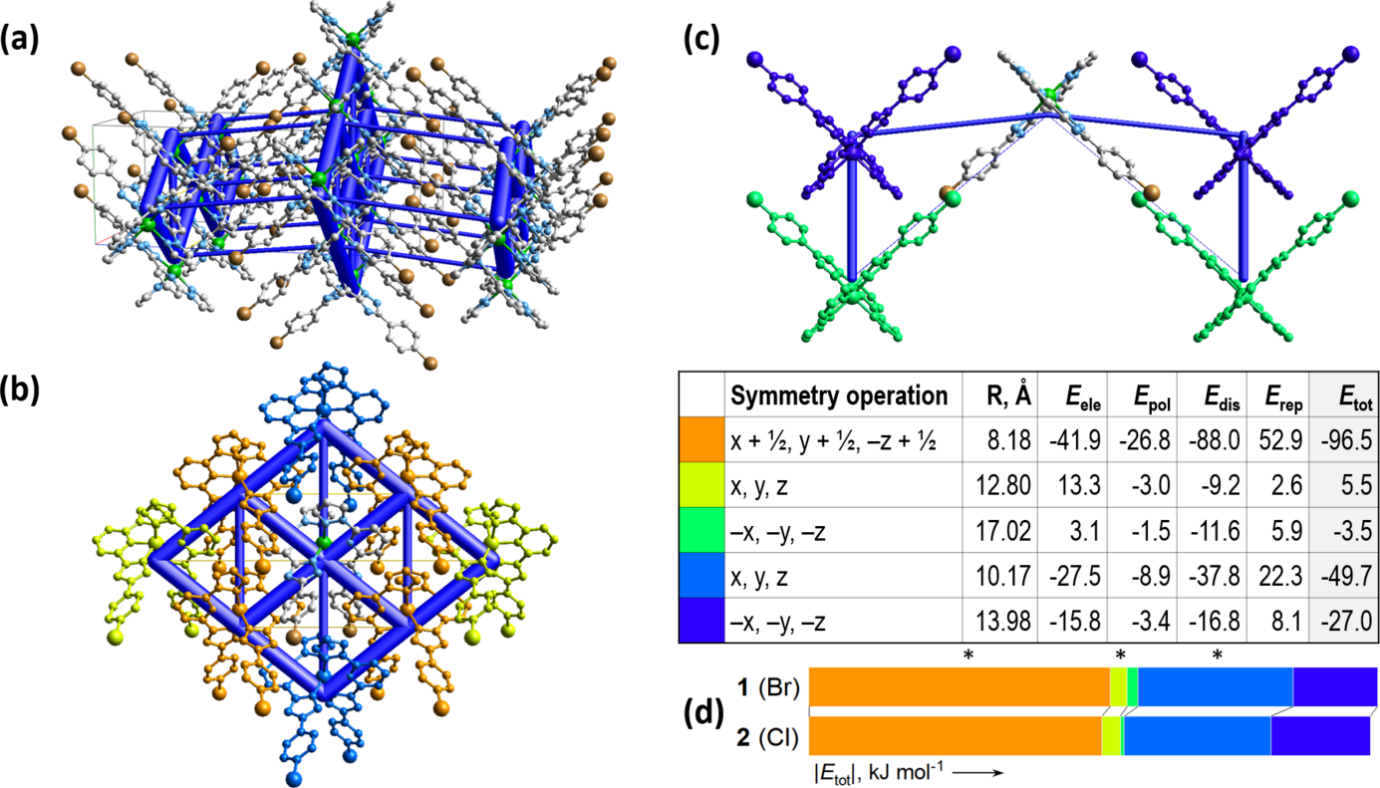
(*a*) The calculated energy frameworks, showing the total energy diagrams (*E*_tot_), (*b*) decomposition of the energy framework into the part corresponding to the inter­actions within a supra­molecular layer and (*c*) inter­layer inter­actions. In the table, the corresponding color-coded energy values *E*_tot_ are provided, including their *E*_ele_, *E*_pol_, *E*_dis_, and *E*_rep_ components. Tube size is set at 100 scale, the blue color corresponds to the attractive inter­actions, yellow to the repulsive inter­actions; (*d*) Comparative plots of the absolute *E*_tot_ values for **1** and **2**. The color-coding of the bars corresponds to the symmetry operations in the table above. The asterisks distinguish the energy bars corresponding to the intra­layer inter­actions.

The energy framework (Spackman *et al.*, 2021 ▸), calculated at the HF/3-21G level, includes electrostatic (*E*_ele_), polarization (*E*_pol_), dispersion (*E*_dis_), repulsion (*E*_rep_) components and total energy (*E*_tot_). Cylindrical radii are scaled to the relative strength. Dispersion forces dominate in the crystal of neutral mol­ecules, and the framework topology reflects the described intra- and inter­layer inter­actions. Calculated *E*_tot_ values are −49.7 kJ mol^−1^ (intra­chain), down to −96.5 kJ mol^−1^ (inter­chain), and −27.0 kJ mol^−1^ (inter­layer). Color-coded inter­action mappings and detailed energy contributions within 3.8 Å of a central mol­ecule are summarized in Fig. 5 ▸*a*–*c*. Fig. 5 ▸*d* presents a bar plot comparing the *E*_tot_ values of **1** and **2**. Despite identical mol­ecular structures and packing arrangements, variations in the size and electronegativity of halogen substituents account for the differing strengths of inter­molecular inter­actions in the two compounds. Consequently, interactions within a supramol­ecular layer are stronger in **1**, whereas the inter­layer inter­actions are comparatively weaker.

## 6. Database survey

A search of the Cambridge Structural Database (CSD, Version 5.42; Groom *et al.*, 2016 ▸) identifies neutral Ni complexes with tridentate bis­azolpyridine ligands containing deprotonable azole groups, such as YOCFAZ (Yuan *et al.*, 2014 ▸), ZOCKOT (Xing *et al.*, 2014 ▸), and ZOTVIP (Wei *et al.*, 2015 ▸). Table 2 ▸ summarizes the structural parameters of these complexes along with complex **2** (KULRIW).

## 7. Synthesis and crystallization

The ligand was synthesized by a modified procedure reported earlier (Seredyuk *et al.*, 2022 ▸), and the synthesis of the title complex followed the method of **2** (Znovjyak *et al.*, 2024 ▸). All chemicals were purchased from commercial suppliers and used without further purification (Merck, Enamine Ltd.).

**3-(4-Bromo­phen­yl)-5-(6-pyrazol­yl(2-pyrid­yl))-1*****H*****-1,2,4-tri­a­zole** (***L***). A Schlenk flask with an inert atmosphere was charged with 6-(1*H*-pyrazol-1-yl)pyridin-2-ylboronic acid, (1.00 g, 5.3 mmol), 5-iodo-3-(4-bromo­phen­yl)-1-(tetra­hydro-2*H*-pyran-2-yl)-1*H*-1,2,4-triazole (2.09 g, 4.8 mmol), [Pd(PPh_3_)_4_] (0.61 g, 0.53 mmol) and Na_2_CO_3_ (1.65 g, 15.6 mmol). Degassed 1,4-dioxane (20 mL) and degassed water (10 mL) were added, and the reaction mixture was heated to 373 K under vigorous stirring for 16 h. After filtering through a Celite pad, to the obtained solution HCl_aq_ (37%, 5 ml) was added dropwise and the obtained solution was stirred for 10 min. Thereafter the pH of the solution was brought to neutral with an aqueous solution of NaOH (10%). The resulting suspension was evaporated to dryness and resuspended in water, and the precipitate was collected by filtration, washed with water and recrystallized from chloro­form-acetone (1:1). After drying *in vacuo*, the final compound was isolated as an analytically pure white crystalline powder. Yield: 1.02 g, 57%. Elemental analysis calculated for C_16_H_11_BrN_6_: C, 52.34; H, 3.02; N, 22.89. Found: C, 52.12; H, 3.11; N, 22.62. ^1^H NMR (300 MHz, 298 K, DMSO-*d*_6_): δ (ppm) 14.90 (1H, *s*, trzH), 9.16 (1H, *s*, pzH), 8.12–7.96 (5H, *m*, phH/pyH), 7.83 (1H, *s*, pzH), 7.64 (2H, *d*, *J* = 8.4 Hz, phH), 6.62 (1H, *s*, pzH). ^13^C NMR (75 MHz, DMSO-*d*_6_): δ (ppm) 161.5, 154.5, 150.9, 144.7, 143.0, 141.4, 132.1, 130.7, 128.7, 128.3, 122.9, 118.8, 113.1, 108.7.

Complex **1** was produced by a layering technique in a standard test tube. The layering sequence was as follows: the bottom layer contained a solution of [Ni(*L*_2_)](ClO_4_)_2_ prepared by dissolving *L* (101 mg, 0.274 mmol) and Ni(ClO_4_)_2_·6H_2_O (50 mg, 0.137 mmol) in boiling acetone, to which chloro­form (5 ml) was then added. The middle layer was a methanol–chloro­form mixture (1:10, 10 ml), which was covered by a layer of methanol (10 ml), to which 5 drops of NEt_3_ were added. The tube was sealed, and light violet plate-like single crystals appeared after 2 weeks (yield *ca.* 65%). Elemental analysis calculated for C_34_H_28_Br_2_N_12_NiO_2_: C, 47.75; H, 3.30; N, 19.65. Found: C, 47.52; H, 3.41; N, 19.73.

## 8. Refinement

Crystal data, data collection and structure refinement details are summarized in Table 3 ▸. H atoms were refined as riding [C—H = 0.95–0.98 Å with *U*_iso_(H) = 1.2–1.5*U*_eq_(C)]. The hydrogen atom H1*A* was refined freely.

## Supplementary Material

Crystal structure: contains datablock(s) I. DOI: 10.1107/S2056989025007467/ee2019sup1.cif

Structure factors: contains datablock(s) I. DOI: 10.1107/S2056989025007467/ee2019Isup2.hkl

Supporting information file. DOI: 10.1107/S2056989025007467/ee2019Isup3.cdx

CCDC reference: 2481668

Additional supporting information:  crystallographic information; 3D view; checkCIF report

## Figures and Tables

**Table 1:** Hydrogen-bond geometry (Å, °) *Cg* is the centroid of the C11–C16 ring.

*D*—H⋯*A*	*D*—H	H⋯*A*	*D*⋯*A*	*D*—H⋯*A*
C3—H3⋯O1^i^	0.95	2.35	3.269 (8)	162
C5—H5⋯O1^i^	0.95	2.48	3.413 (7)	167
C1—H1⋯N6^ii^	0.95	2.30	3.238 (6)	171
C7—H7⋯C1^iii^	0.95	2.71	3.615 (7)	161
O1—H1*A*⋯N5	0.73 (6)	2.08 (6)	2.798 (6)	168 (6)
C2—H2⋯*Cg*^iv^	0.95	2.69	3.542 (6)	140

Symmetry codes: (i) 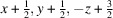; (ii) 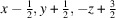; (iii) 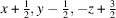; (iv) 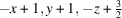.

**Table 2:** Computed distortion indices for the title compound and for similar complexes reported in the literature

CSD refcode	<*M*—N> (Å)	*Σ* (°)	*Θ* (°)	CShM(O_h_)
**1**	2.097	119.3	386.9	3.70
**2**_(KULRIW)	2.095	119.4	387.3	3.71
YOCFAZ	2.088^*a*^	120.8^*a*^	397.6^*a*^	3.65^*a*^
ZOCKOT	2.086	121.0	375.9	3.78
ZOTVIP	2.110	124.9	382.4	3.55

Note: (*a*) averaged value.

**Table 3:** Experimental details

Crystal data	
Chemical formula	[Ni(C_16_H_10_BrN_6_)_2_]·2CH_4_O
*M*_r_	855.21
Crystal system, space group	Orthorhombic, *Pbcn*
Temperature (K)	200
*a*, *b*, *c* (Å)	12.8038 (8), 10.1729 (4), 27.9377 (14)
*V* (Å^3^)	3638.9 (3)
*Z*	4
Radiation type	Mo *K*α
μ (mm^−1^)	2.78
Crystal size (mm)	0.3 × 0.2 × 0.03

Data collection	
Diffractometer	Xcalibur, Eos
Absorption correction	Multi-scan (*CrysAlis PRO*; Rigaku OD, 2024 ▸)
*T*_min_, *T*_max_	0.534, 1.000
No. of measured, independent and observed [*I* > 2σ(*I*)] reflections	11620, 3218, 2074
*R*_int_	0.064
(sin θ/λ)_max_ (Å^−1^)	0.595

Refinement	
*R*[*F*^2^ > 2σ(*F*^2^)], *wR*(*F*^2^), *S*	0.061, 0.134, 1.04
No. of reflections	3218
No. of parameters	236
H-atom treatment	H atoms treated by a mixture of independent and constrained refinement
Δρ_max_, Δρ_min_ (e Å^−3^)	0.74, −0.81

Computer programs: *CrysAlis PRO* 1.171.43.124 (Rigaku OD, 2024 ▸), *SHELXT* (Sheldrick, 2015*a* ▸), *SHELXL2018/3* (Sheldrick, 2015*b* ▸) and *OLEX2* (Dolomanov *et al.*, 2009 ▸).

## supplementary crystallographic information

### Bis{3-(4-bromophenyl)-5-[6-(1H-pyrazol-1-yl)pyridin-2-yl]-4H-1,2,4-triazol-4-ido}nickel(II) methanol disolvate Crystal data

**Table:**

[Ni(C_16_H_10_BrN_6_)_2_]·2CH_4_O	*D*_x_ = 1.561 Mg m^−^^3^
*M**_r_* = 855.21	Mo *K*α radiation, λ = 0.71073 Å
Orthorhombic, *P**b**c**n*	Cell parameters from 1992 reflections
*a* = 12.8038 (8) Å	θ = 2.6–21.9°
*b* = 10.1729 (4) Å	µ = 2.78 mm^−^^1^
*c* = 27.9377 (14) Å	*T* = 200 K
*V* = 3638.9 (3) Å^3^	Plate, clear colourless
*Z* = 4	0.3 × 0.2 × 0.03 mm
*F*(000) = 1720	

### Bis{3-(4-bromophenyl)-5-[6-(1H-pyrazol-1-yl)pyridin-2-yl]-4H-1,2,4-triazol-4-ido}nickel(II) methanol disolvate Data collection

**Table:**

Xcalibur, Eos diffractometer	3218 independent reflections
Radiation source: fine-focus sealed X-ray tube, Enhance (Mo) X-ray Source	2074 reflections with *I* > 2σ(*I*)
Graphite monochromator	*R*_int_ = 0.064
Detector resolution: 16.1593 pixels mm^-1^	θ_max_ = 25.0°, θ_min_ = 2.2°
ω scans	*h* = −8→15
Absorption correction: multi-scan (CrysAlisPro; Rigaku OD, 2024)	*k* = −12→12
*T*_min_ = 0.534, *T*_max_ = 1.000	*l* = −33→23
11620 measured reflections	

### Bis{3-(4-bromophenyl)-5-[6-(1H-pyrazol-1-yl)pyridin-2-yl]-4H-1,2,4-triazol-4-ido}nickel(II) methanol disolvate Refinement

**Table:**

Refinement on *F*^2^	Primary atom site location: dual
Least-squares matrix: full	Hydrogen site location: mixed
*R*[*F*^2^ > 2σ(*F*^2^)] = 0.061	H atoms treated by a mixture of independent and constrained refinement
*wR*(*F*^2^) = 0.134	*w* = 1/[σ^2^(*F*_o_^2^) + (0.042*P*)^2^ + 6.2503*P*] where *P* = (*F*_o_^2^ + 2*F*_c_^2^)/3
*S* = 1.03	(Δ/σ)_max_ < 0.001
3218 reflections	Δρ_max_ = 0.74 e Å^−^^3^
236 parameters	Δρ_min_ = −0.81 e Å^−^^3^
0 restraints	

### Bis{3-(4-bromophenyl)-5-[6-(1H-pyrazol-1-yl)pyridin-2-yl]-4H-1,2,4-triazol-4-ido}nickel(II) methanol disolvate Special details

**Table:**

Geometry. All esds (except the esd in the dihedral angle between two l.s. planes) are estimated using the full covariance matrix. The cell esds are taken into account individually in the estimation of esds in distances, angles and torsion angles; correlations between esds in cell parameters are only used when they are defined by crystal symmetry. An approximate (isotropic) treatment of cell esds is used for estimating esds involving l.s. planes.

### Bis{3-(4-bromophenyl)-5-[6-(1H-pyrazol-1-yl)pyridin-2-yl]-4H-1,2,4-triazol-4-ido}nickel(II) methanol disolvate Fractional atomic coordinates and isotropic or equivalent isotropic displacement parameters (Å^2^)

**Table:**

	*x*	*y*	*z*	*U*_iso_*/*U*_eq_	
Br1	0.66307 (8)	0.02071 (7)	0.48878 (3)	0.0797 (4)	
Ni1	0.500000	0.70049 (8)	0.750000	0.0216 (2)	
N1	0.5117 (3)	0.8456 (4)	0.80613 (15)	0.0256 (10)	
N2	0.6139 (3)	0.8692 (4)	0.81923 (16)	0.0248 (10)	
N3	0.6556 (3)	0.7082 (4)	0.76558 (14)	0.0219 (10)	
N4	0.5627 (3)	0.5623 (4)	0.70264 (15)	0.0231 (10)	
N5	0.5333 (3)	0.4789 (4)	0.66679 (15)	0.0251 (10)	
N6	0.7063 (3)	0.4515 (4)	0.67934 (16)	0.0259 (10)	
C1	0.4561 (4)	0.9206 (5)	0.83493 (19)	0.0301 (13)	
H1	0.381986	0.925804	0.834432	0.036*	
C2	0.5201 (5)	0.9917 (5)	0.8664 (2)	0.0390 (16)	
H2	0.498267	1.051486	0.890526	0.047*	
C3	0.6200 (5)	0.9572 (5)	0.8552 (2)	0.0335 (14)	
H3	0.681998	0.988952	0.869887	0.040*	
C4	0.6923 (4)	0.7938 (5)	0.79686 (18)	0.0241 (12)	
C5	0.7978 (4)	0.8087 (5)	0.80651 (19)	0.0325 (14)	
H5	0.822350	0.871958	0.828859	0.039*	
C6	0.8649 (4)	0.7276 (5)	0.7822 (2)	0.0377 (15)	
H6	0.938019	0.735362	0.787377	0.045*	
C7	0.8278 (4)	0.6343 (5)	0.7501 (2)	0.0349 (14)	
H7	0.874470	0.577053	0.733845	0.042*	
C8	0.7215 (4)	0.6267 (5)	0.74235 (18)	0.0237 (12)	
C9	0.6658 (4)	0.5433 (5)	0.70830 (18)	0.0237 (12)	
C10	0.6212 (4)	0.4151 (5)	0.65395 (19)	0.0260 (13)	
C11	0.6278 (4)	0.3207 (5)	0.61459 (19)	0.0275 (13)	
C12	0.7116 (5)	0.2340 (5)	0.6123 (2)	0.0380 (15)	
H12	0.762148	0.235364	0.637192	0.046*	
C13	0.7239 (5)	0.1457 (6)	0.5749 (2)	0.0470 (17)	
H13	0.782299	0.088134	0.573719	0.056*	
C14	0.6488 (5)	0.1440 (5)	0.5396 (2)	0.0403 (16)	
C15	0.5639 (5)	0.2259 (5)	0.5405 (2)	0.0414 (16)	
H15	0.513185	0.222718	0.515651	0.050*	
C16	0.5530 (5)	0.3147 (5)	0.57862 (19)	0.0345 (14)	
H16	0.494019	0.371228	0.579850	0.041*	
O1	0.3537 (4)	0.5660 (5)	0.61938 (19)	0.0542 (14)	
H1A	0.397 (5)	0.548 (6)	0.635 (2)	0.05 (2)*	
C17	0.3843 (6)	0.6447 (6)	0.5808 (3)	0.070 (2)	
H17A	0.404825	0.588562	0.553908	0.105*	
H17B	0.443564	0.699591	0.590398	0.105*	
H17C	0.325855	0.700940	0.571141	0.105*	

### Bis{3-(4-bromophenyl)-5-[6-(1H-pyrazol-1-yl)pyridin-2-yl]-4H-1,2,4-triazol-4-ido}nickel(II) methanol disolvate Atomic displacement parameters (Å^2^)

**Table:**

	*U*^11^	*U*^22^	*U*^33^	*U*^12^	*U*^13^	*U*^23^
Br1	0.1213 (9)	0.0692 (5)	0.0486 (5)	0.0147 (5)	−0.0015 (5)	−0.0307 (4)
Ni1	0.0135 (5)	0.0257 (5)	0.0254 (5)	0.000	−0.0008 (5)	0.000
N1	0.016 (3)	0.031 (2)	0.030 (2)	0.001 (2)	−0.006 (2)	0.0002 (19)
N2	0.014 (2)	0.028 (2)	0.032 (3)	0.0003 (19)	−0.002 (2)	−0.005 (2)
N3	0.016 (2)	0.022 (2)	0.028 (2)	−0.0013 (19)	−0.001 (2)	−0.0033 (18)
N4	0.018 (3)	0.026 (2)	0.025 (2)	−0.0010 (19)	−0.001 (2)	−0.0036 (18)
N5	0.019 (3)	0.030 (2)	0.026 (2)	−0.003 (2)	0.000 (2)	−0.0039 (19)
N6	0.017 (2)	0.028 (2)	0.033 (3)	−0.0011 (19)	0.003 (2)	−0.0058 (19)
C1	0.022 (3)	0.037 (3)	0.031 (3)	0.005 (3)	0.002 (3)	0.001 (2)
C2	0.037 (4)	0.045 (4)	0.035 (3)	0.010 (3)	0.006 (3)	−0.013 (3)
C3	0.027 (3)	0.037 (3)	0.036 (3)	0.000 (3)	−0.006 (3)	−0.015 (3)
C4	0.019 (3)	0.023 (3)	0.030 (3)	0.003 (2)	−0.004 (3)	−0.002 (2)
C5	0.024 (3)	0.041 (3)	0.032 (3)	−0.002 (3)	−0.006 (3)	−0.014 (3)
C6	0.016 (3)	0.050 (4)	0.047 (4)	0.001 (3)	−0.007 (3)	−0.010 (3)
C7	0.018 (3)	0.044 (3)	0.043 (4)	0.003 (3)	0.001 (3)	−0.015 (3)
C8	0.019 (3)	0.029 (3)	0.023 (3)	−0.001 (2)	0.000 (3)	0.000 (2)
C9	0.015 (3)	0.029 (3)	0.027 (3)	−0.002 (2)	−0.002 (3)	0.000 (2)
C10	0.025 (3)	0.025 (3)	0.028 (3)	−0.005 (2)	−0.001 (3)	0.001 (2)
C11	0.029 (3)	0.027 (3)	0.026 (3)	−0.003 (2)	0.004 (3)	0.001 (2)
C12	0.039 (4)	0.041 (3)	0.033 (3)	0.003 (3)	−0.007 (3)	−0.008 (3)
C13	0.050 (5)	0.046 (4)	0.045 (4)	0.014 (3)	0.006 (4)	−0.006 (3)
C14	0.062 (5)	0.028 (3)	0.031 (3)	0.002 (3)	0.006 (4)	−0.006 (2)
C15	0.053 (4)	0.042 (4)	0.028 (3)	−0.005 (3)	−0.004 (3)	−0.003 (3)
C16	0.037 (4)	0.029 (3)	0.038 (3)	−0.001 (3)	−0.003 (3)	0.002 (3)
O1	0.032 (3)	0.073 (3)	0.058 (3)	−0.004 (3)	−0.009 (3)	0.026 (3)
C17	0.094 (7)	0.054 (4)	0.063 (5)	−0.014 (4)	−0.021 (5)	0.015 (4)

### Bis{3-(4-bromophenyl)-5-[6-(1H-pyrazol-1-yl)pyridin-2-yl]-4H-1,2,4-triazol-4-ido}nickel(II) methanol disolvate Geometric parameters (Å, º)

**Table:**

Br1—C14	1.903 (5)	C5—H5	0.9500
Ni1—N1^i^	2.159 (4)	C5—C6	1.372 (7)
Ni1—N1	2.159 (4)	C6—H6	0.9500
Ni1—N3	2.041 (4)	C6—C7	1.390 (7)
Ni1—N3^i^	2.041 (4)	C7—H7	0.9500
Ni1—N4^i^	2.091 (4)	C7—C8	1.380 (7)
Ni1—N4	2.091 (4)	C8—C9	1.461 (7)
N1—N2	1.380 (5)	C10—C11	1.462 (7)
N1—C1	1.318 (6)	C11—C12	1.390 (7)
N2—C3	1.347 (6)	C11—C16	1.389 (7)
N2—C4	1.409 (6)	C12—H12	0.9500
N3—C4	1.320 (6)	C12—C13	1.386 (8)
N3—C8	1.349 (6)	C13—H13	0.9500
N4—N5	1.366 (5)	C13—C14	1.378 (8)
N4—C9	1.344 (6)	C14—C15	1.370 (8)
N5—C10	1.348 (6)	C15—H15	0.9500
N6—C9	1.339 (6)	C15—C16	1.404 (7)
N6—C10	1.352 (6)	C16—H16	0.9500
C1—H1	0.9500	O1—H1A	0.73 (6)
C1—C2	1.403 (7)	O1—C17	1.398 (8)
C2—H2	0.9500	C17—H17A	0.9800
C2—C3	1.364 (7)	C17—H17B	0.9800
C3—H3	0.9500	C17—H17C	0.9800
C4—C5	1.386 (7)		
			
N1—Ni1—N1^i^	93.7 (2)	C6—C5—C4	116.7 (5)
N3—Ni1—N1^i^	101.33 (16)	C6—C5—H5	121.7
N3^i^—Ni1—N1	101.33 (16)	C5—C6—H6	119.5
N3^i^—Ni1—N1^i^	75.58 (16)	C5—C6—C7	121.0 (5)
N3—Ni1—N1	75.58 (16)	C7—C6—H6	119.5
N3^i^—Ni1—N3	175.6 (2)	C6—C7—H7	120.8
N3—Ni1—N4	77.64 (16)	C8—C7—C6	118.5 (5)
N3^i^—Ni1—N4	105.42 (16)	C8—C7—H7	120.8
N3^i^—Ni1—N4^i^	77.64 (16)	N3—C8—C7	120.4 (5)
N3—Ni1—N4^i^	105.42 (16)	N3—C8—C9	111.5 (5)
N4^i^—Ni1—N1^i^	153.21 (16)	C7—C8—C9	128.0 (5)
N4—Ni1—N1	153.21 (16)	N4—C9—C8	118.2 (4)
N4—Ni1—N1^i^	91.54 (16)	N6—C9—N4	114.2 (4)
N4^i^—Ni1—N1	91.54 (16)	N6—C9—C8	127.6 (5)
N4^i^—Ni1—N4	95.5 (2)	N5—C10—N6	113.6 (4)
N2—N1—Ni1	112.2 (3)	N5—C10—C11	124.4 (5)
C1—N1—Ni1	143.3 (4)	N6—C10—C11	121.9 (5)
C1—N1—N2	104.5 (4)	C12—C11—C10	119.7 (5)
N1—N2—C4	117.6 (4)	C16—C11—C10	122.2 (5)
C3—N2—N1	111.6 (4)	C16—C11—C12	118.1 (5)
C3—N2—C4	130.6 (5)	C11—C12—H12	118.9
C4—N3—Ni1	120.9 (3)	C13—C12—C11	122.2 (6)
C4—N3—C8	120.2 (4)	C13—C12—H12	118.9
C8—N3—Ni1	118.9 (3)	C12—C13—H13	121.1
N5—N4—Ni1	140.9 (3)	C14—C13—C12	117.9 (6)
C9—N4—Ni1	113.6 (3)	C14—C13—H13	121.1
C9—N4—N5	105.5 (4)	C13—C14—Br1	118.4 (5)
C10—N5—N4	105.3 (4)	C15—C14—Br1	119.3 (5)
C9—N6—C10	101.3 (4)	C15—C14—C13	122.3 (5)
N1—C1—H1	124.3	C14—C15—H15	120.6
N1—C1—C2	111.4 (5)	C14—C15—C16	118.9 (6)
C2—C1—H1	124.3	C16—C15—H15	120.6
C1—C2—H2	127.1	C11—C16—C15	120.6 (5)
C3—C2—C1	105.8 (5)	C11—C16—H16	119.7
C3—C2—H2	127.1	C15—C16—H16	119.7
N2—C3—C2	106.7 (5)	C17—O1—H1A	112 (5)
N2—C3—H3	126.6	O1—C17—H17A	109.5
C2—C3—H3	126.6	O1—C17—H17B	109.5
N3—C4—N2	113.6 (4)	O1—C17—H17C	109.5
N3—C4—C5	123.2 (5)	H17A—C17—H17B	109.5
C5—C4—N2	123.2 (5)	H17A—C17—H17C	109.5
C4—C5—H5	121.7	H17B—C17—H17C	109.5
			
Br1—C14—C15—C16	178.8 (4)	C1—N1—N2—C4	175.0 (4)
Ni1—N1—N2—C3	−178.2 (3)	C1—C2—C3—N2	−0.6 (6)
Ni1—N1—N2—C4	−3.0 (5)	C3—N2—C4—N3	174.0 (5)
Ni1—N1—C1—C2	176.8 (4)	C3—N2—C4—C5	−6.4 (9)
Ni1—N3—C4—N2	3.7 (6)	C4—N2—C3—C2	−173.9 (5)
Ni1—N3—C4—C5	−175.9 (4)	C4—N3—C8—C7	−1.7 (7)
Ni1—N3—C8—C7	176.4 (4)	C4—N3—C8—C9	−178.1 (4)
Ni1—N3—C8—C9	0.1 (5)	C4—C5—C6—C7	−0.9 (9)
Ni1—N4—N5—C10	−177.8 (4)	C5—C6—C7—C8	1.4 (9)
Ni1—N4—C9—N6	177.7 (3)	C6—C7—C8—N3	−0.1 (8)
Ni1—N4—C9—C8	−5.4 (6)	C6—C7—C8—C9	175.6 (5)
N1—N2—C3—C2	0.5 (6)	C7—C8—C9—N4	−172.4 (5)
N1—N2—C4—N3	−0.2 (6)	C7—C8—C9—N6	4.0 (9)
N1—N2—C4—C5	179.4 (5)	C8—N3—C4—N2	−178.2 (4)
N1—C1—C2—C3	0.5 (7)	C8—N3—C4—C5	2.2 (8)
N2—N1—C1—C2	−0.2 (6)	C9—N4—N5—C10	0.6 (5)
N2—C4—C5—C6	179.5 (5)	C9—N6—C10—N5	−0.8 (6)
N3—C4—C5—C6	−0.9 (8)	C9—N6—C10—C11	175.8 (5)
N3—C8—C9—N4	3.6 (6)	C10—N6—C9—N4	1.2 (6)
N3—C8—C9—N6	180.0 (5)	C10—N6—C9—C8	−175.3 (5)
N4—N5—C10—N6	0.1 (6)	C10—C11—C12—C13	−177.6 (5)
N4—N5—C10—C11	−176.4 (4)	C10—C11—C16—C15	177.7 (5)
N5—N4—C9—N6	−1.2 (6)	C11—C12—C13—C14	−1.0 (9)
N5—N4—C9—C8	175.7 (4)	C12—C11—C16—C15	−1.7 (8)
N5—C10—C11—C12	−162.1 (5)	C12—C13—C14—Br1	−178.7 (4)
N5—C10—C11—C16	18.4 (8)	C12—C13—C14—C15	0.1 (9)
N6—C10—C11—C12	21.6 (8)	C13—C14—C15—C16	0.0 (9)
N6—C10—C11—C16	−157.8 (5)	C14—C15—C16—C11	0.9 (8)
C1—N1—N2—C3	−0.2 (6)	C16—C11—C12—C13	1.8 (9)

Symmetry code: (i) −*x*+1, *y*, −*z*+3/2.

### Bis{3-(4-bromophenyl)-5-[6-(1H-pyrazol-1-yl)pyridin-2-yl]-4H-1,2,4-triazol-4-ido}nickel(II) methanol disolvate Hydrogen-bond geometry (Å, º)

*Cg* is the centroid of the C11–C16 ring.

**Table:**

*D*—H···*A*	*D*—H	H···*A*	*D*···*A*	*D*—H···*A*
C3—H3···O1^ii^	0.95	2.35	3.269 (8)	162
C5—H5···O1^ii^	0.95	2.48	3.413 (7)	167
C1—H1···N6^iii^	0.95	2.30	3.238 (6)	171
C7—H7···C1^iv^	0.95	2.71	3.615 (7)	161
O1—H1*A*···N5	0.73 (6)	2.08 (6)	2.798 (6)	168 (6)
C2—H2···*Cg*^v^	0.95	2.69	3.542 (6)	140

Symmetry codes: (ii) *x*+1/2, *y*+1/2, −*z*+3/2; (iii) *x*−1/2, *y*+1/2, −*z*+3/2; (iv) *x*+1/2, *y*−1/2, −*z*+3/2; (v) −*x*+1, *y*+1, −*z*+3/2.

### Hydrogen-bond geometry (Å, °)

**Table:**

*D–H···A*	*D–H*	*H···A*	*D···A*	*D–H···A*
C3–H3···O1^i^	0.95	2.35	3.268 (7)	162
C5–H5···O1^i^	0.95	2.48	3.410 (7)	167
C1–H1···N6^ii^	0.95	2.30	3.238 (6)	171
C7–H7···C1^ii^	0.95	2.71	3.615 (7)	161
O1–H1A···N5	0.73 (6)	2.08 (6)	2.798 (6)	168 (6)

Symmetry codes: (i) 1/2 + x, 1/2 + y, 1.5 - z; (ii) 1/2 + x, -1/2 + y, 1.5 - z
